# Knowledge and Awareness of HPV Vaccine and Acceptability to Vaccinate in Sub-Saharan Africa: A Systematic Review

**DOI:** 10.1371/journal.pone.0090912

**Published:** 2014-03-11

**Authors:** Stacey Perlman, Richard G. Wamai, Paul A. Bain, Thomas Welty, Edith Welty, Javier Gordon Ogembo

**Affiliations:** 1 Pathfinder International, Watertown, Massachusetts, United States of America; 2 Northeastern University, Boston, Massachusetts, United States of America; 3 Countway Library of Medicine, Harvard Medical School, Boston, Massachusetts, United States of America; 4 Cameroon Baptist Convention Health Services, Bamenda, Cameroon; 5 University of Massachusetts Medical School, Worcester, Massachusetts, United States of America; Universidad Nacional de La Plata, Argentina

## Abstract

**Objectives:**

We assessed the knowledge and awareness of cervical cancer, HPV and HPV vaccine, and willingness and acceptability to vaccinate in sub-Saharan African (SSA) countries. We further identified countries that fulfill the two GAVI Alliance eligibility criteria to support nationwide HPV vaccination.

**Methods:**

We conducted a systematic review of peer-reviewed studies on the knowledge and awareness of cervical cancer, HPV and HPV vaccine, and willingness and acceptability to vaccinate. Trends in Diphtheria-tetanus-pertussis (DTP3) vaccine coverage in SSA countries from 1990–2011 were extracted from the World Health Organization database.

**Findings:**

The review revealed high levels of willingness and acceptability of HPV vaccine but low levels of knowledge and awareness of cervical cancer, HPV or HPV vaccine. We identified only six countries to have met the two GAVI Alliance requirements for supporting introduction of HPV vaccine: 1) the ability to deliver multi-dose vaccines for no less than 50% of the target vaccination cohort in an average size district, and 2) achieving over 70% coverage of DTP3 vaccine nationally. From 2008 through 2011 all SSA countries, with the exception of Mauritania and Nigeria, have reached or maintained DTP3 coverage at 70% or above.

**Conclusion:**

There is an urgent need for more education to inform the public about HPV, HPV vaccine, and cervical cancer, particularly to key demographics, (adolescents, parents and healthcare professionals), to leverage high levels of willingness and acceptability of HPV vaccine towards successful implementation of HPV vaccination programs. There is unpreparedness in most SSA countries to roll out national HPV vaccination as per the GAVI Alliance eligibility criteria for supporting introduction of the vaccine. In countries that have met 70% DTP3 coverage, pilot programs need to be rolled out to identify the best practice and strategies for delivering HPV vaccines to adolescents and also to qualify for GAVI Alliance support.

## Introduction

The introduction of vaccines has been one of the most effective public health interventions for combating infectious diseases [1], [2]. The establishment of the Expanded Programme on Immunization (EPI) in 1974 by the World Health Organization (WHO) led to the global eradication of smallpox and has greatly reduced the burden of several infectious diseases, including poliomyelitis, measles, tuberculosis, tetanus and diphtheria in many parts of the world [2]. Despite slow progress in increasing vaccine access and immunization coverage, the EPI has reported 83% coverage of infants worldwide of the three doses of Diphtheria-tetanus-pertussis (DTP3) vaccine in 2011, similar to coverage in 2009 (82%) and 2010 (85%) [1], [3]. Expansion and delivery of life-saving vaccines in the 2010–2020 “decade of vaccines” is expected to save 6.4 million lives, valued at hundreds of billions of dollars in low and middle-income countries [4]. Currently, DTP3 coverage by age 12 months is a key indicator of immunization program performance of a country and is associated with the level of capacity to effectively manage and deliver a new vaccine to a target cohort [5], [6].

Worldwide, 15% of all cancer cases and nearly 26% of cancer cases in developing countries are attributable to infectious agents, particularly viruses [7]. Cervical cancer, which is caused by the human papillomavirus (HPV), is the leading cause of cancer mortality among women in sub-Saharan Africa (SSA) [8], [9]. The approval and recommendation of two vaccines – Gardasil and Cervarix – provide a huge opportunity to curb the burden of cervical cancer [10]. As one of the key strategies in preventing cervical cancer in developed countries, providing HPV vaccines in low and middle income countries is a critical pillar for meeting the global action plan for closing the cancer divide [5]. However, outstanding barriers to achieving this goal in low-income countries remain. These include high cost of vaccine and vaccine delivery [11], low cervical cancer screening levels [12], poor health system capabilities [11], [13], inaccessibility to medical care [14], low awareness and knowledge of HPV and cervical cancer[14]–[18], and failure of cervical cancer to be recognized as a major health concern [19].

Several recent developments have emphasized HPV vaccine as an important prevention strategy. The 2009 WHO position paper on HPV vaccines recommended they be included in routine national immunization programs as a public health priority [20]. Furthermore, one of the goals of the 2006 Global Immunization Vision and Strategy (GIVS) is to introduce new vaccines to all eligible populations within five years of introduction in national programs [3]. Additionally, major milestones during 2007–2011 have brought access to HPV vaccines within reach for many adolescents in low-income countries. In May 2013, for the first time ever, a public offer was made by GAVI Alliance for a price of $4.50 per dose for both Gardasil and Cervarix to low-income countries [21], a drastic reduction from $360 for the required three doses [22], [23].

Currently, the GAVI Alliance uses two criteria to determine eligibility for vaccination support, including HPV vaccine: 1) a DTP3 threshold of 70% national coverage (WHO/UNICEF estimates) and 2) a pilot demonstration of the ability to deliver a complete multi-dose series of vaccines to at least 50% of the target vaccination cohort in an average sized district in a country [24]. Recently, Rwanda was the first country to take advantage of the low pricing through a partnership with pharmaceutical manufacturer Merck, achieving 93% coverage of HPV vaccination of all grade six adolescent girls in 2011 [25]–[27], which is, to the best of our knowledge, the highest in the world. Through other sources of subsidized HPV vaccines, 88.9% of girls were fully vaccinated in Uganda using a school-based pilot program supported by PATH International in 2009 [28]. A similar HPV vaccination pilot initiative was recently undertaken in Cameroon [15], Tanzania [29], Lesotho [30] and South Africa [31]. More recently, Kenya became the first SSA country to receive GAVI Alliance support to roll out a HPV vaccine pilot project (see: http://www.gavialliance.org/support/nvs/human-papillomavirus-vaccine-support). GAVI Alliance also announced that in 2014 it will support the first nationwide introduction of HPV vaccine in Rwanda for girls of all eligible ages, as well as other HPV demonstration projects in Mozambique, Zimbabwe, Ghana, Madagascar, Malawi, Niger, Sierra Leone, and Tanzania (see: http://www.gavialliance.org/support/nvs/human-papillomavirus-vaccine-support).

These developments, coupled with SSA's recent success in reaching 70% coverage for other routine vaccines, namely measles, hepatitis B, Influenza, tuberculosis and polio since 1990 [3], provide strong evidence of how introducing HPV vaccine can be achieved in other countries meeting the GAVI Alliance eligibility criteria. The success of pilot demonstrations in Rwanda, South Africa, Cameroon, Lesotho, Tanzania and Uganda also provide lessons on how to design and implement national HPV vaccination programs tailored for a specific group of individuals in resource-limited regions.

Nevertheless, introducing HPV vaccine in SSA offers unique challenges, especially due to limited awareness of cervical cancer, its relationship to HPV, concerns about safety and future fertility, and political factors [32], as seen in recent cases in Rwanda [26], [27] and Cameroon [33]. These unsubstantiated rumors about side effects or adverse outcomes that may not be casually related to the vaccine may negatively impact public trust and adversely impact HPV immunization programming leading to suspension of the program altogether as recently experienced in Japan [34] and India [35].

In this study, we assessed the knowledge and awareness of cervical cancer, HPV and HPV vaccine, willingness and acceptability to vaccinate through a systematic review of peer-reviewed literature. We further identified the fulfillment of GAVI Alliance eligibility criteria among countries in SSA. To the best of our knowledge this is the first systematic review of the potential readiness for introduction of HPV vaccine in the sub-continent, which reveals further insight into some of the unique challenges that need to be addressed.

## Methods

### Identification of Studies

Studies examining awareness, knowledge of cervical cancer, HPV and acceptability of HPV vaccines in SSA were identified by searching PubMed/MEDLINE (NCBI), Embase (Elsevier), African Index Medicus (AIM), and POPLINE (K4Health) from their earliest dates through July 11, 2013. Bibliographies of relevant reviews and eligible studies were examined for additional sources. The search was performed by a librarian (PAB) using terms for papillomavirus vaccines and vaccination, acceptability or awareness, and the names of all SSA countries (Appendix S1). The search was conducted without language restriction. Titles and abstracts of identified articles were reviewed by two authors (SP and JGO) and categorized as relevant using the criteria outlined below.

The review was conducted using methodology reported in the National Health Service Centre for Reviews and Dissemination report, supplemented by Harden's recommendations for systematic reviews of qualitative studies [36]. We also adhered to guidance on methods for conducting and reporting systematic reviews in the PRISMA statement where it could be applied to mixed method reviews [37]. Two authors (JGO and RGW) appraised the quality of the studies using a checklist developed by the National Institute for Health and Clinical Excellence [38], and surveys using a checklist adapted from Pettigrew and Roberts [39] provided in supplementary Table 1 and Table 2, respectively.

**Table 1:** A summary of countries included in the systematic review.

Total Number of Articles	29
Total Number of Studies	27
Total Number of Countries	13
Botswana	1
Cameroon	5
Ghana	1
Kenya	2
Lesotho	1
Mali	1
Nigeria	5
Rwanda	1
South Africa	4
Tanzania	4
Uganda	3
Zambia	1
Zimbabwe	1
Demographics of Studies* *Note: one study looked at multiple demographics	Girls (Pupils)– 6
	Medical Professionals – 8
	HCW – 3
	Gyn – 1
	Nurses – 3
	Non-traditional healers – 1
	Parents – 5
	Women – 10
	University students – 1
	Age 12–26 – 1
	Age 12–84 – 1
	Age 15–49 – 1
	Age 16–64 – 1
	Age 18–44 – 2
	Age 18–65 – 2
	Women from HIV-1 discordant couples – 1
	Not Applicable/Unspecific – 2
Methods	Survey – 17
	Focus Groups – 2
	School-based Vaccination – 2
	Assessment of Vaccination Programs – 1
	Case Control Study – 1
	Cross-Sectional Study of Delivery Strategies - 1
	Discussion/Interviews – 1
	KAP Studies – 1
	Randomized Controlled Trials – 1
	Review – 1
Acceptability of HPV Vaccine (12 Studies) *Note – 1 study assessed two different vaccine delivery strategies, resulting in two different levels of acceptability; *1 study looked at the results of vaccination strategies in two different countries, resulting in two different levels of acceptability. [14], [15], [25], [28], [30], [31], [41], [42], [53]–[55]	High – 12
	Moderate - 2
	Low – 0
Acceptability of cervical cancer Screening (1 study) [56]	High – 1
	Low – 0
Willingness to Recommend HPV Vaccines (5 Studies) [15], [17], [43], [57], [58]	High – 5
	Low – 0
Willingness to Get Vaccinated (4 Studies) [42], [43], [53], [59]	High – 4
	Low – 0
Willingness to Get Daughter Vaccinated (4 Studies) [14], [17], [40], [53]	High – 4
	Low – 0
Willingness to Participate in Trials (1 Study) [60]	High – 1
	Low – 0
Interest in Vaccine for Daughters (1 Study) [61]	High – 1
	Low – 0
Interest in Learning More about Vaccine (1 Study) [62]	High – 1
	Low – 0
Knowledge of Cervical Cancer, HPV and/or HPV Vaccine (16 Studies) [14], [29], [42], [56]–[67]	High – 0
	Moderately High - 1
	Moderate - 2
	Low – 16
	None – 3
Awareness of CC, HPV and/or HPV Vaccine (15 Studies) [14], [15], [17], [40]–[43], [53], [55]–[57], [59], [61], [64], [67]	High – 11
	Low – 9
	Moderate – 2

**Table 2:** Overview of knowledge and awareness of HPV, cervical cancer and HPV vaccine, willingness to vaccinate, and acceptability of HPV vaccine in sub-Saharan Africa*.

Study, Country	Demographic	Sample Size/Method	Level of Knowledge/Awareness of HPV, CC and Vaccine	Acceptability/Willingness	Strategies for Vaccination/Increasing Awareness	Factors Influencing Acceptability
[25] **Rwanda**	Girls – Primary school	93,888 (1st round vaccination) School-based vaccination & Community Sensitization	Not discussed	High Acceptability - Achieved 93.23% coverage after first three-dose course of vaccination	School-based Vaccination; Nationwide population sensitization campaign implemented months in advance of vaccination.	Not discussed
[31] **South Africa**	Girls – Primary School (9–12 years)	1,926 (1^st^ round vaccination); School-based vaccination strategy)	Not discussed	High Acceptability – 97.8% coverage after three-dose course of vaccination	School-based vaccination strategy used/highly recommended; Awareness, information and education sessions on cervical cancer, screening, and an HPV demonstration project were conducted with school principals, governing bodies and teachers.	Not discussed
[30] **Cameroon**	Girls - (9–18 years)	1,033 girls vaccinated out of 1,600 targeted girls; Assessment of Gardasil Access Program: Health facility vaccination site	Not discussed	Moderate acceptability – 64.5% estimated program coverage	Health facility vaccination strategy; More in-depth discussion sessions with parents and caregivers, evaluations of the knowledge of and attitudes toward HPV vaccination in these audiences will be important to the success of future HPV vaccination campaigns.	Not discussed
[30] **Lesotho**	Girls – (9–18 years old)	33,818 girls vaccinated out of 40,100 girls targeted; Assessment of Gardasil Access Program: Mixed vaccination strategy (clinic-based and school-based)	Not discussed	High acceptability - 71.4% estimated program coverage	Overall results of the 8 vaccination programs indicate that school-based strategies were most effective for reaching girls aged 9–13 years while mixed models had better overall performance compared with models using just one of the methods.	Not discussed
[29] **Tanzania**	Girls – Primary School	5,532 eligible for vaccination: 3,352 for class-based delivery; 2,180 for age-based delivery; Randomized. Control Trial: Phase IV cluster-randomized trial of 2 vaccine delivery strategies: age-based (targeting girls born in 1998) and class-based (targeting girls in school class 6)	Not discussed	High Acceptability – Achieved 76.1% coverage of all three doses (total of age-based and class-based delivery strategies)	School-based vaccination divided into two delivery strategies: age-based and class-based. A sensitization campaign was conducted prior to vaccination by providing teachers, parents/guardians and girls with verbal and written information about HPV vaccination through multiple formats.	Head teachers at 3 private schools randomized to the age-based strategy, with an estimated 25 eligible pupils in total, would not permit vaccination, fearing negative parental feedback.
[68] **Tanzania**	Pupils/Adults	404 Case Control Study of HPV vaccine receivers and non-receivers	Low knowledge of cervical cancer among girls; Moderate knowledge of HPV vaccine among girls - “Vaccine specifically mentioned” as cervical cancer prevention - (52.8% among cases; 74.3% among controls)	High acceptability - see Watson-Jones et al. 2012a	School-based vaccination	Acceptance of vaccine among adult controls: Prevention of cervical cancer was the primary reason. Other reasons included health benefits, knowing someone who had cancer and encouragement by the project team. Among pupil controls: protection of cervical cancer, health benefits, parental wishes. Rejection of vaccine among adult cases: concerns over side effects or infertility; insufficient knowledge about the vaccine; daughter was absent from school on the vaccination day. Rejection of vaccine among pupil cases: they had been absent from school the day of vaccination; both parents had refused permission for vaccination; concerns about side effects; afraid of injections; infertility concerns. Sensitization messages and parent meetings are critical for vaccine acceptance while persistent concerns about vaccine side effects and potential impact of fertility need to be closely addressed in a national vaccine program. It is important to give parents and pupils time to reconsider their decisions about vaccination – those who initially did not accept vaccination would have done so if offered another opportunity.
[57] **Cameroon**	Healthcare workers	401; Survey: cross-sectional, self-administered	High awareness - cervical cancer; Low knowledge about cervical cancer among nurses & midwives; Low knowledge about HPV vaccine among Healthcare workers (exception among gynecologists).	High willingness to recommend among those who believe HPV vaccine can prevent cancer.* *Low belief that HPV vaccine prevents cancer: Only 44%.	Continuing medical education programs including nurse-midwives should be conducted at hospital level to spread knowledge about cervical cancer prevention.	Not discussed
[67] Oral Presentation **Nigeria**	Healthcare workers - Gynecologists	118; Survey: cross-sectional	High awareness - HPV, cervical cancer and HPV vaccine; Low knowledge - eligibility and schedule of HPV vaccine	Not discussed	87.4% suggested incorporation into national immunization program; 34.5% agreed vaccination should be precondition for school enrollment; 16.1% agreed client should pay for vaccine.	Not discussed
[55] **Nigeria**	Healthcare workers (female)	177; Survey: cross-sectional	High awareness of cervical cancer; High awareness of HPV; Moderate awareness of HPV vaccine	High acceptability of HPV vaccine (defined as willingness to recommend to their adolescent daughters, other adolescents or sexually unexposed daughters	Necessary strategies: public enlightenment, subsidize cost and improve access/availability. There is a need for public enlightenment, which have the potential of creating awareness and improving acceptability and uptake of most health-care programs.	Not discussed
[62] **South Africa**	Healthcare workers – nontraditional healers known as Sangomas	12; Focus Groups	Low knowledge – HPV; No knowledge – HPV vaccine; Moderate knowledge – Cervical Cancer (“somewhat familiar”)	Not discussed but after learning about HPV vaccine, participants were excited about the possibility to prevent cervical cancer. There was high desire to learn more.	Participants stressed the important of educating parents, especially fathers about HPV. HPV and cervical cancer prevention workshops are important and useful tools for educating the public. Collaborate/engage with Sangomas to develop culturally appropriate prevention strategies given they have great access to community members.	Not discussed
[54] **Tanzania**	Healthcare workers, Teachers, Parents, Female Pupils & Religious Leaders	169; Discussion & Interviews: Qualitative sub-study using group discussions and in-depth interviews	Almost no knowledge - Cervical Cancer, HPV or HPV vaccine; Only healthcare workers had knowledge but low	High acceptability: high acceptability to receive HPV vaccine among pupils; high acceptability of HPV vaccine for daughters among rest.	Positive views on intensive sensitization to increase awareness from all respondents. School-based delivery programs need adequate sensitization to prevent past instances of rumors undermining such campaigns. Respondents in favor of age-based vaccination.	Parents - “Prevention is better than cure”; Girls - Avoid dangerous disease like cervical cancer; Most participants - Trusted safety of vaccine.
[58] **Cameroon**	Nurses	76; Survey: Exploratory	Low levels of knowledge about HPV infection, symptoms and prevention of cervical cancer; Moderately high level of knowledge about HPV vaccine	High level of willingness: Two-thirds (69.7%) would recommend HPV vaccine to targeted 9–13 year old girls	Recommend intensive on-the-job continuing education program for current nursing staffs and other health care workers regarding the importance of cervical cancer screening, HPV as an STI and the role of HPV vaccine in the prevention of cervical cancer. Training should be systematically included in nurses' education curricula so newly trained nurses can effectively promote and provide HPV immunizations when they are universally available.	Not discussed
[64] **Tanzania**	Nurses	137; Survey: Cross sectional questionnaire	Low knowledge - cervical cancer; Low awareness/knowledge - HPV vaccine	Not discussed	Health promotion and disease prevention policies, awareness campaigns and screening programs. Integration of screening services into existing programs.	Not discussed
[43] **Nigeria**	Nurses (female)	178; Survey: Cross-sectional, descriptive	High awareness - cervical cancer and HPV; Low knowledge - HPV infection; Low awareness & Knowledge - HPV vaccine	High willingness to be vaccinated; High willingness to recommend	A well-designed HPV education program integrated into a national cervical cancer prevention and control program is needed to bridge information gap	Reason for **rejection** to recommend to adolescents: Main reason given: Insufficient knowledge about vaccine & complications. Other reasons given: Too youngNot yet sexually activeNot at risk for HPVVaccination encourages promiscuity. No reasons discussed for nurses accepting vaccine for themselves
[17] **Cameroon**	Parents	337; Survey: Cross-sectional	High awareness - cervical cancer, HPV, HPV vaccine	High willingness for daughters; High willingness to recommend	Recommended community-based sensitization to increase awareness	In order highest to lowest: Effectiveness, Side effects, Healthcare provider recommendation, Cost
[40] **Botswana**	Parents/Adults	372; Survey: Cross-sectional	High Awareness – cervical cancer; Low Awareness - HPV/HPV Vaccine	High willingness for daughters	Willingness to get daughters vaccinated at schools - programs in Botswana should consider schools as potential venues. Spread information by addressing several health belief model constructs associated with high acceptability.	Belief that cervical cancer and genital warts were serious health problems (perceived severity)l; Access to vaccine; Recommendation from doctor (lower if from nurse)
[28] **Uganda**	Parents/Guardians	1,489; Cross-sectional study: three delivery strategies (School-based strategy and school-based strategy combined w/Child Days Plus Programme in Uganda)	Not discussed	High acceptability - 90.5% coverage in year 1 and 88.9% coverage in year 2, through school-based strategy; Moderate acceptability −52.6% in year 1 and 60.7% in year 2, vaccination coverage through School-based combined w/Child Days Plus.	School-based Vaccination and School-based Vaccination Combined w/Child Days Plus Programme.	Protection against cervical cancer; Prevention of disease; Vaccines thought good for health or wanted girl to be healthy.
[69] Continuation of LaMontagne et al. 2011 **Uganda**	Parents/Guardians	1,489; Cross-sectional study	Not discussed	High acceptability – overall acceptability for two different vaccination strategies (school-based and school-based combined with Child Days Plus) based on LaMontagne et al. 2011.	School-based Vaccination and School-based Vaccination Combined w/Child Days Plus Programme.	Exposure to community influencers was an important factor when parents made decisions about HPV vaccination for their young adolescent daughters. Exposure to information, education and communication (IEC) materials only marginally increased vaccine uptake; it is the people with whom parents and other guardians speak, and not the type of IEC materials and activities they are exposed to, that facilitates uptake of the HPV vaccine. HPV vaccination programs should focus on comprehensive. communication strategies that utilize key community influencers and stakeholders as opposed to targeted delivery of IEC materials and activities.
[42] **Nigeria**	Women (university students)	375; Survey: Cross-sectional	Low awareness – HPV; Moderate awareness – cervical cancer; Low knowledge – cervical cancer risk factors	High willingness; High acceptability	Public health education efforts through community and media involvement to ensure acceptance and uptake - educational approaches through radio, television and folk media; vaccine advocacy	Reasons for **rejection**: Fear of side-effects; Fear of the unknown; Controversies surrounding vaccines. Predictors of Acceptability: Age; Medical Education; Knowledge of HPV/Awareness of cervical cancer.
[15] **Cameroon**	Women (12–26) Adolescents	553; Survey: Cross-sectional survey	High awareness - HPV, cervical cancer and HPV Vaccine	High acceptability for themselves; High willingness to recommend	Recommended community-based sensitization to increase awareness	Not discussed
[56] **Zimbabwe**	Women (12–84) Rural	514; Survey: Descriptive	Low awareness/knowledge - Cervical cancer and cervical cancer screening	High acceptability - cervical cancer screening after educational intervention	Government should recognize cervical cancer as major public health concern; mass education on preventing STIs, targeting adolescents and young adults. Education curricula of nurses and physicians should incorporate promotion of cervical cancer screening and treatments to increase awareness. The HPV vaccine should be incorporated into the Zimbabwe Expanded Programme on Immunization (ZEPI); Dialogues should be initiated among community representatives to dispel myths of immunizations.	Not discussed
[61] **Kenya**	Women (15–49)	147; Survey: Questionnaires administered by interviewers	Limited awareness - cervical cancer and cervical cancer screening; No knowledge - HPV vaccine	High interest for daughters; Not willing to pay for vaccine	Basic education campaign is necessary for introduction of HPV vaccine to achieve optimal prevention	Main factors influencing women to vaccinate their daughter: If it protected against cervical cancer and free (95%); If it protected against cervical cancer and genital warts and free (95%); If it protected against genital warts and free (94%); If it required only one shot (86%).
[65] **Nigeria**	Women (16–64)	198; Survey: Cross-sectional	Low knowledge – Cervical cancer; No knowledge – HPV vaccine	Not discussed	There is a need to establish an intensive and sustainable awareness campaign on preventing cervical cancer	Not discussed
[14] **South Africa**	Women (18–44)	86; Survey	High awareness - cervical cancer; Low awareness – HPV; Low knowledge - HPV, cervical cancer; *Women unfamiliar w/HPV, cervical cancer and HPV vaccine	High willingness for daughters if recommended by provider	Recommendation to develop prevention and education messages and social marketing campaigns to increase awareness.	46% of women surveyed were very likely to vaccinate based on provider recommendation; Most important factor when deciding to vaccinate from highest to lowest): Unsure/Do not know; Provider Recommendation; Side Effects; Effectiveness; Cost
[66] **South Africa**	Women (18–44)	24; Focus Groups	Limited knowledge about HPV, cervical cancer and HPV vaccine	High acceptability for children - once vaccine explained* *Acceptability - contingent upon request for more information/education	Focus on primary prevention strategies such as culturally appropriate multigenerational educational materials for girls, mothers and grandmothers; Provide access to prevention education, screening, treatment and obtain knowledge needed to make informed reproductive health decisions.	Main factor influencing acceptability was keeping their children safe and protect them. The majority of women felt they were okay with the vaccine as long as someone explains what the shot is for and how it will help their children.
[41] **Ghana**	Women (18–65)	264; Survey: Based on the Health Belief Model	High awareness - cervical cancer; Low awareness - HPV vaccine	High acceptability for themselves & daughters	Schools are ideal venue for vaccine distribution; Community health education programs which are best directed through clear school health education programs, public TV health announcements.	Perceived risk for cervical cancer (themselves & daughters);Access to vaccine; Social support for vaccine use.
[53] **Zambia**	Women (18–65)	319; Survey: Cross-sectional	High awareness – cervical cancer	High willingness to vaccinate themselves and daughters; High acceptability of HPV vaccine	Health clinics were cited as most often as women's source of information regarding vaccination, making clinics a logical venue for cervical cancer education.	Cost was the only acceptability factor examined. Only 47.4% would pay something for the vaccine.
[59] **Kenya**	Women from HIV-1-discordant couples	409; Survey: Questionnaire administered by clinical staff	High awareness – HPV; Low Knowledge – HPV; Low awareness – HPV vaccine	High willingness to be vaccinated	There is a need for education surrounding cause and prevention of cervical cancer – not just for vaccination but also for Pap screening and self-sampling.	Cost – willing to vaccinate if available at little or no cost.
[60] **Mali**	Unspecified	51; Knowledge, Attitudes, Practices (KAP) Studies	Low Knowledge - HPV	High Willingness to Participate in vaccine trials	Not discussed	Not discussed
[70] **Uganda**	Not applicable	Review of opportunities and obstacles	Not applicable	Not applicable	Opportunities for Universal Routine Vaccination: Political commitment; Enabling laws and policies for strengthening health systems; Create vaccine acceptability; Create partnerships through orgs like GAVI Alliance; Build synergies between HPV vaccination and existing health programs. A School-based strategy and an age-based strategy were used in different regions and proved effective.	Not discussed - This was not looked at since there was no sample surveyed. However, it did reference other studies noting that there is high acceptability in the country and reasons for non-acceptance were driven by programmatic considerations such as school absenteeism rather than opposition.

### Inclusion Criteria

Studies included in the review needed to meet the following criteria: 1) it occurred in a SSA country; 2) it was published in 2006 or later, after HPV vaccination was introduced; 3) it focused on one or more of the three key demographics (adolescents, parents/guardians, healthcare workers); and 4) it examined at least one or more of the following key themes: a) level of awareness of HPV and/or cervical cancer; b) level of knowledge of cervical cancer and/or awareness; c) willingness to vaccinate; d) and acceptability of HPV vaccine. Studies that conducted a pilot HPV vaccination program were automatically included in the review. Animal studies were excluded from the review.

### Article Review

A systematic review of the identified studies was then performed summarizing key results. The authors' findings were treated as primary data and studies were synthesized using a framework approach. Data were extracted and organized by key information such as demographic, method and sample size, and key findings of the study about levels of knowledge, awareness, willingness and acceptability. Any strategies for vaccination or increasing awareness (whether recommended or implemented strategies) were also extracted along with factors influencing acceptability of HPV vaccine.

### Analysis

Levels of awareness, knowledge, acceptability and willingness were broken down into these categories: 1) awareness of cervical cancer, HPV and/or HPV vaccine; 2) knowledge of cervical cancer, HPV and/or HPV vaccine; 3) acceptability of HPV vaccine (and in one case, acceptability of cervical cancer screening); and 4) willingness to vaccinate or get vaccinated. Willingness was broken down further into categories based on how each individual article defined it as seen in Table 1. Levels of awareness, knowledge, acceptability and willingness to vaccinate were summarized based on how each individual study categorized the levels (high, low or moderate). The articles were then tallied for each relevant category. Some articles had multiple levels of awareness and knowledge, resulting in one article being counted more than once (i.e. one article may have high awareness of cervical cancer but low awareness of HPV). Strategies for vaccination/increasing awareness and factors influencing acceptability provided qualitative insight and allowed us to identify important themes for awareness, implementation and acceptability of HPV vaccination.

## Results

### Overview of Studies Examined

The literature search returned 142 unique records as summarized in the flow chart (Fig. S1). Review of selected article bibliographies uncovered 10 other articles. A total of 124 relevant articles were selected and reviewed and 29 articles based on 27 studies in 13 different SSA countries met the inclusion criteria. Ten studies focused on women, with ages ranging from 12 to 84, eight studies focused on medical professionals, five focused on parents and six focused on girls in primary schools. Six countries implemented HPV vaccination pilot programs (Cameroon, Lesotho, Rwanda, South Africa, Tanzania and Uganda). Table 1 shows a detailed summary of identified studies and Table 2 summarizes the key findings from each study.

### Study Quality

Two qualitative studies were considered to be of good standard while one was considered moderate and one was considered poor. Nearly all of the surveys were well conducted and 17 of these had a sample size greater than 200 respondents. Sixteen of those had a response rate greater than 70%. The remaining studies varied in quality from moderately good to poor [30].

### Awareness and Knowledge of Cervical Cancer and HPV

Fifteen studies examined awareness of cervical cancer, HPV and/or HPV vaccine among specific demographic groups. Levels of awareness were mixed with 11 studies demonstrating high awareness, nine studies demonstrating low awareness and two studies demonstrating moderate awareness. Levels of knowledge of cervical cancer and HPV were consistently low. Of the 16 studies examining knowledge of cervical cancer, HPV and HPV vaccine, all noted low levels of knowledge, three reported no knowledge, and two reported moderate knowledge. Only one study specified a moderately high level of knowledge of HPV vaccine.

### Willingness and Acceptability of HPV Vaccine and Cervical Cancer Screening

Categories of willingness varied across studies: willingness to recommend HPV vaccine (five studies); willingness to get vaccinated (four); willingness to get daughter vaccinated (four); willingness to participate in vaccine trials (one); “interest” in the vaccine for daughters (one); and “interest in learning more about the vaccine” (one). All studies reported high rates of willingness in their respective categories. Twelve studies examined acceptability levels of HPV vaccine and one study examined acceptability of cervical cancer screening (Table 1). All 12 studies reported high levels of acceptability of HPV vaccine. However, multiple levels of acceptability were found within the studies because some assessed and compared different vaccine delivery strategies within and in different countries.

### Strategies and Factors for Increasing Awareness and Acceptability of HPV Vaccine

Of the 27 studies, 26 discussed strategies for vaccination and increasing awareness. Studies in the six countries in the review where HPV vaccination pilot programs were conducted discussed the implementation strategies used for vaccination while the remaining countries in this review discussed recommended strategies for implementation. Rwanda, South Africa Tanzania and Uganda used school-based strategies achieving 93.2%, 97.8%, 71.6% and 88.9% vaccine uptake, respectively [25], [28]. Lesotho used a mixed vaccination strategy (clinic and school-based) [30]. Cameroon used three distinct approaches to deliver vaccines namely school-based, health facilities and community outreach programs in churches and mother-to-daughter [15], [17], [18]. In addition, all studies, with the exception of Lesotho specifically mentioned that sensitization campaigns were used in their vaccination strategies [30]. Rwanda conducted a nationwide campaign while South Africa, Tanzania, Cameroon and Uganda conducted focused educational campaigns to targeted groups [15], [25], [28], [29], [31]. Two studies in both Botswana [40] and Ghana [41] recommended schools as the ideal venue for HPV vaccine delivery.

Education for increasing awareness was a strong theme throughout the majority of studies. Recommended strategies to implement sensitization programs included community health education programs, continuing medical education for nurses, midwives, doctors and other healthcare workers, and health promotion and policy programs including awareness through social and mass media (i.e. public radio, television and folk media).

Factors influencing acceptability also varied. Twelve studies addressed reasons for acceptability of HPV vaccine. Two studies in Nigeria [42], [43] discussed reasons for rejection among nurses and university students while one study in Tanzania [29] noted that head teachers at three private schools would not permit vaccination, fearing negative parental feedback. In Nigeria [43], nurses' reasons included: insufficient knowledge about HPV vaccine; girls were too young and not sexually active; girls are not yet at risk to HPV infections; and it encourages promiscuity. In addition, university students who rejected the HPV vaccine based their decisions on fear of side effects, fear of the unknown, and controversies surrounding the vaccine [42]. Themes surrounding acceptability included access to the vaccine, side effects and effectiveness, protection against and prevention of cervical cancer, provider and teacher recommendations, support from the National Immunization Program and cost [42], [43].

### GAVI Alliance Eligibility Based on DTP3 Levels and Pilot Demonstration Projects on HPV Vaccine Delivery

Of the 13 countries included in this review, 12 have achieved 70% coverage or higher of the DTP3 vaccine since 2003, one of GAVI Alliance's criteria for vaccine support; the exception is Nigeria (Table 3). Six of those countries had an HPV vaccination pilot program (Cameroon, Lesotho, Rwanda, South Africa, Tanzania and Uganda). There were 31 sub-Saharan African countries not included in the review because there were no studies based in these countries that met the inclusion criteria. The majority of these countries did not start consistently reaching coverage levels of 70% or higher until 2005 (Table 4). From 2008 through 2011, all countries, with the exception of Mauritania, have maintained coverage at 70% or above (Table 4).

**Table 3:** Reported Estimates of DTP3 Coverage in sub-Saharan Countries included in this review.

Country	2011	2010	2009	2008	2007	2006	2005	2004	2003	2002	2001	2000	1999	1998	1997	1996	1995	1994	1993	1992	1991	1990
**Botswana**	99	95	97	94	98	99	99	89	93	87	74	85	85	82	76	83	80	78	57	59	53	56
* **Cameroon**	82	84	80	84	82	81	80	73	73	63	43	53	48	48	43	44	46	38	34	37	34	36
**Ghana**	91	94	94	93	94	84	84	80	80	99	76	84	72	68	60	51	51	48	48	40	40	50
**Kenya**	88	83	75	85	81	80	76	73	73	84	80	63	79	64	36	77	84	50	42	40	41	42
* **Lesotho**	69	75	72	91	91	90	87	71	62	60	72	69	58	64	56	53	52	55	47	66	67	71
**Mali**	88	92	89	99	91	95	95	86	79	74	61	54	47	56	52	53	49	39	46	38	34	42
**Nigeria**	61	74	71	57	69	72	38	38				38		21	21	26	34	44	29	43	39	56
* **Rwanda**	97	97	97	97	97	99	95	89	96	88	77	90	85		77	98	90		83	85	89	57
* **South Africa**	97	91	99	98	97	99	97	89	94	82	81	96	76	76	73	73	72		81	79	81	74
* **Tanzania**	92	91	85	86	83	90	90	95	95	89	87	79	76	79	79	82	81	84	83	83	81	78
* **Uganda**	82	80	83	79	85	80	84	87	81	72	61	58	60	56	61	72	74	79	73	71	77	77
**Zambia**	81	84	98	95	92	97	91	94	91	88		76	92		70	83	82	86	67	61	91	71
**Zimbabwe**	93	89	73	75	85	90	90	85	80	75	75	77	81	70	78	80	83	80	73	79	89	78

Source: WHO http://apps.who.int/immunization_monitoring/globalsummary/timeseries/tscoveragedtp3.html.

*Country with HPV Vaccination Pilot Program.

**Table 4:** Reported Estimates of DTP3 Coverage in Countries Not Included in the Review.

Country Name	2011	2010	2009	2008	2007	2006	2005	2004	2003	2002	2001	2000	1999	1998	1997	1996	1995	1994	1993	1992	1991	1990
Angola	86	91	73	81	83	44	47	59	46	47	41	31	22	45	41	28	42	27	30	21	26	24
Benin	99	98	98	93	97	93	93	83	88	93	84	88	90	81	78	80	89	86	77	79	68	78
Burkina Faso	91	91	99	99	99	95	96	88	84	69	68	57	34	40	70	48	47	41	47	39		
Burundi	99	96	99	92	99	92	87	83	94	95	59	68	63	50	60	55	63	48	63	80	83	86
Cape Verde	90	99	74	82	81	72	73	75	87	94	78	86	69	80	78	73	73		99	99	99	88
Central African Republic (the)	64	58	76	51	84	88	46	50	28	23	23	29	27	45	53	53	45	40		31	66	61
Chad	70	83	75	43	70	77	58	50	47	40	27	28	33	23	24	20	18	18	13	10	18	20
Comoros (the)	83	74	83	81	75	69	68	76	80	89	70	70		75	48	60	75	58	70	38	52	94
Congo (the)	90	90	91	89	80	79	65	67	50	41	31	33	29		23	75	47		60	65	70	77
Côte d'Ivoire	62	85	81	74	76	77	56	50	48	73	59	62	58	64	70	55	41	41	50	49	54	
Democratic Republic of the Congo (the)	90	78	92	83	87	77	73	64	49	43	32	40	25	18	18	18	23	29	29		16	36
Equatorial Guinea	54	44	74	74	41	34	34	46	41	65	32	32	40		81	64	64		60	64		
Eritrea	94	85	85	85	80	80	80	80	75	70	65	52	56	60	60	46	35	36	28			
Ethiopia	87	86	79	81	73	72	69	66	52	51	51	42	40	37	41	42	57	37	28	13	21	49
Gabon	75	67	76	82	81	44	40	40	40	33	28	10	31	37	54	61	70	59	65	66	72	78
Gambia (the)	96	95	94	96	94	93	89	92	90	80	96	74	88	97	96	96	96	93	90	85	85	92
Guinea	85	90	85	70	93	89	86	69	69	58	57	57	57	56	53	48	73	73		52	41	20
Guinea-Bissau	89	86	82	79	96	77	80	80	77	50	47		6		63	53	45	74	65	67	63	61
Liberia	77	75	92	92	88	88	87	31	38	51	62	48	23	19	26	45	45					
Madagascar	89	85	89	88	95	93	92	75	87	62	37	80	71	54	61	73	74	66	66	65	53	71
Malawi	97	93	93	91	87	99	93	89	84	64	90	75	85	96	95	90	97	98	91	86	81	80
Mauritania	75	64	64	74	75	68	71	70	76	83	61	31	26		28	50	50	50	44	39	29	33
Mauritius	98	99	99	99	97	97	97	98	92		93	88	85	89	90	89	93	89		88		85
Mozambique	76	74	76	80	75	98	75	65	85	84	80	88	81	77	61	60	57	55	49	50	46	46
Namibia	82	83	83	83	86	74	86	81	92	77	63	79	72	74	66	70	74	79	73	70	87	59
Sao Tome and Principe	96	98	98	99	97	99	97	99	94	91	92	82		73	73	68	79		60	68	87	92
Senegal	83	70	86	88	94	89	84	87	73	60	52	52	60	59	59	70	80	57	52	46	51	66
Seychelles	99	99	99	99	99	99	99	99	99	99	96		99	99	98	99	98	96		96		99
Sierra Leone	76	76	91	87	79	95	64	61	70	52	38	24		56	26	65	43	41	63	64	56	83
Swaziland	91	89	72	80	68	68	71	83	95	77	77	98	99	84	82	82	82	96	89	93	79	89
Togo	92	92	89	89	88	87	82	71	72	59	43	50	48	36	40	27	47	71	75	76	82	77

Source: WHO http://apps.who.int/immunization_monitoring/en/globalsummary/timeseries/tscoveragedtp3.htm.

## Discussion

This review identified low levels of knowledge and mixed levels of awareness on cervical cancer, HPV and HPV vaccine, and high levels of acceptability of HPV vaccine among all key demographics. In particular, high acceptability of HPV vaccine despite the lack of knowledge about cervical cancer and HPV represents an opportunity for increased education and awareness strategies about cervical cancer, HPV and HPV vaccine. This is important to help key demographics understand the transmission of HPV, its characteristics and associated risks [17], [44], and the benefits of HPV vaccine.

Engaging all key demographics through improved and increased education will elevate public trust, which is a critical component of successful implementation of widespread vaccine coverage. Incidences such as those seen in Japan, India and Rwanda [26], [27], [34], have challenged public trust in HPV vaccines. Additionally, the qualitative insight provided by this review shows that factors influencing acceptability are often tied to issues of public trust, such as concerns over side effects and safety. This is not only a concern in low- and mid-income countries. For instance, Japan's Ministry of Health recently withdrew its recommendation for administering HPV vaccine because of reports of side effects, although it still pays for the vaccine for parents consenting to immunize their daughters [34].

Anti-vaccination groups frequently post inaccurate information about vaccine side effects on the web, which is publicized by both local and national media [46], [47]. These examples indicate the need to constantly engage and educate the public to avoid the risk of health programs failing [48], [49]. While it may not be advisable or possible to respond to all such misinformation, it is essential to counteract it by providing scientifically correct information in a proactive manner so people will seek appropriate medical advice for clarification [50].

Tailored community-based interventions and sensitization programs are a viable means to achieve this for multiple reasons. They have the potential to curb concerns about safety and effectiveness of the vaccine while dispelling negative myths or controversies [45]. Specific training for healthcare workers, the first contact point for patients, will provide them with the accurate knowledge and information necessary to discuss cervical cancer, HPV and HPV vaccine with their patients as well as the ability to properly detect, screen and test for HPV and cervical cancer [18], [43].

In addition to understanding levels of knowledge, awareness and acceptability, this review uncovered encouraging trends concerning SSA countries' eligibility for GAVI Alliance support. Only six countries have currently met the two criteria required by the GAVI Alliance to support introduction of the HPV vaccine at a lower cost of US$4.50 per dose [21]. Of the six, Rwanda has already achieved 93.2% vaccine coverage among girls in grade six and is taking the lead to enroll all eligible girls for HPV vaccination through GAVI alliance support in 2014.

Further, our analysis of national vaccine coverage for DTP3 in SSA shows rapid expansion from just one country reaching 80% in 1980 to 35 countries in 2010 [6]. Of the 31 countries that did not meet the review inclusion criteria, only Mauritania did not consistently achieve GAVI Alliance's eligibility criteria requiring DTP3 coverage levels greater than or equal to 70% (Table 4). Of the countries included in this review, only Nigeria failed to meet this threshold. The challenges in Nigeria are perhaps unique especially in the context of the recent polio vaccine boycott and killings [51]. This calls for interrogating vaccine acceptance determinants using, for instance, the health-belief model [45] and addressing these through a re-emphasis on contextualized public communication and education to mitigate resistance and to build trust [6].

Such consistently high DTP3 coverage levels among the majority of SSA countries indicates structural health system capabilities to deliver simple vaccines to the population [1], [3]. This further demonstrates the feasibility for these countries to deliver multi-dose vaccines, such as HPV, for 50% of a target vaccination cohort in an average size district, which would qualify them for GAVI Alliance support. Support from GAVI Alliance is a critical and essential gateway to preventing cervical cancer through vaccination in SSA and bridging the global cancer divide [5].

As this review shows, engaging all stakeholders at multiple levels in countries that meet these requirements is necessary for successful HPV vaccine implementation. Governments should leverage the high levels of acceptability and willingness to vaccinate by increasing education for healthcare workers, women/girls and parents/guardians. Governments should also determine appropriate strategies for disseminating information and vaccine delivery by building upon current infrastructures, such as existing EPIs and school-based programs. This has been shown to be an effective approach because it relies on the involvement of multiple critical stakeholders from the government down to the community level, as seen in Rwanda, South Africa, Uganda and Tanzania [2], [6], [25], [26], [28], [31]. Tailored community-based sensitization campaigns aimed at a targeted population, as used in Cameroon, also proved effective [15], [17], [18]. Thus, using hybrid delivery system models may be more beneficial as per country experiences in the EPI and can help inform and determine best practices when developing HPV vaccine pilot programs [4], [6].

### Conclusions

The objective of this study was to provide a systematic review of knowledge and awareness of HPV vaccine, willingness to vaccinate, and acceptability of the vaccine, as well as fulfillment of GAVI Alliance's eligibility criteria for vaccine assistance in SSA. To the best of our knowledge, this is the first systematic review of the potential readiness for introduction of HPV vaccine in the sub-continent. Examining the region collectively offers insight into its readiness and ability to implement HPV vaccination on a broader scale while shedding light on the successes, challenges, and lessons of implementation.

From this review, three important themes have emerged. (1) There are high levels of acceptability and willingness to vaccinate. These should be harnessed by national governments to establish and implement HPV vaccination strategies that build upon existing infrastructures. (2) Overall, six SSA countries qualify for GAVI Alliance assistance to introduce HPV vaccine at a national level. (3) Lastly, there is a need for increased education and awareness among all three key demographics about HPV, HPV vaccine and the burden of cervical cancer as a disease.

The combination of required DTP3 coverage, high acceptability of HPV vaccine and high willingness to vaccinate, indicates the readiness and potential for SSA countries to introduce HPV vaccine to the population. Successfully doing so will depend on implementing tailored delivery strategies that fit each country's needs and engaging the government at all levels. Building upon the lessons learned from GAVI Alliance eligible SSA countries will pave the way for those SSA countries still working to meet the criteria. These countries should also take advantage of and benefit from the “decade of vaccines” and the GIVS when there is a re-dedication by donors to meet these goals [4], [6].

### Limitations

There were some limitations in this review. The studies reviewed lacked consistency in regards to psychometric characteristics used. Not all of the studies used a theoretical framework, consistent labeling of themes examined, or rigorous testing and validation of the measures as previously outlined [52]. While some assessed levels of knowledge and awareness of cervical cancer and HPV, others did not. Still others discussed levels of willingness to vaccinate and acceptability of HPV vaccine while others did not. Some studies discussed all themes. This indicates a need for more standardized methods on awareness and knowledge of HPV and HPV vaccine, and acceptability and willingness to vaccinate to provide better insight to guide health care practitioners in developing successful community and clinical interventions and scaling up HPV vaccination. Every effort was made to include all studies but it is possible some were missed.

## Supporting Information

Figure S1 **Flow Chart Diagram.**
(TIFF)

Table S1 **Quality appraisal of qualitative studies.**
(DOCX)

Table S2 **Quality assessment of surveys.**
(DOCX)

Checklist S1 **PRISMA Checklist**
(DOC)

Appendix S1 **Search strategies for bibliographic databases.**
(DOCX)
