# Supplementary figures and images for: Knowledge and Awareness of HPV Vaccine and Acceptability to Vaccinate in Sub-Saharan Africa: A Systematic Review
Source: PLoS One. 2014 Mar 11;9(3):e90912. doi: 10.1371/journal.pone.0090912 (PMC3949716; doi:10.1371/journal.pone.0090912)

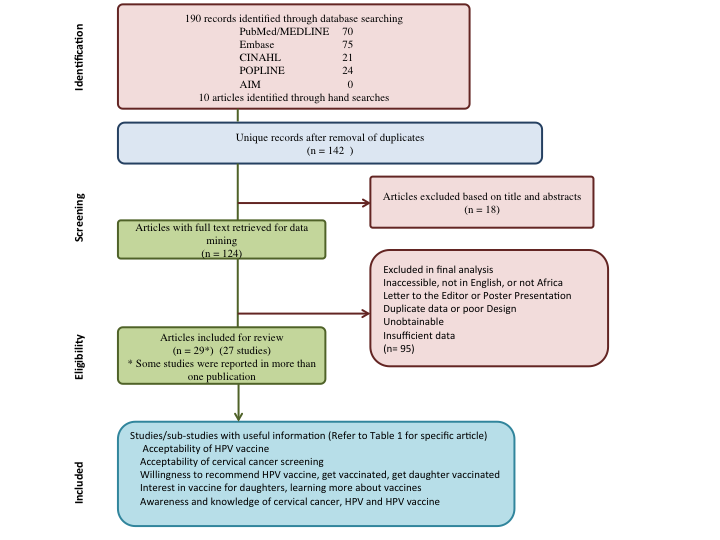

Supplement: Figure S1 — Flow Chart Diagram. (TIFF) [file pone.0090912.s001.tiff]
