# Supplementary material for: Knowledge and Awareness of HPV Vaccine and Acceptability to Vaccinate in Sub-Saharan Africa: A Systematic Review
Source: PLoS One. 2014 Mar 11;9(3):e90912. doi: 10.1371/journal.pone.0090912 (PMC3949716; doi:10.1371/journal.pone.0090912)
Supplement: Table S1 — Quality appraisal of qualitative studies. (DOCX) [file pone.0090912.s002.docx]

**Supplementary Table 1. Quality appraisal of qualitative studies**

|  |  | Banura et al., 2012 | Francis et al., 2011 | Nelson et al., 2010 | Remes et al. 2012 |
| --- | --- | --- | --- | --- | --- |
| **Theoretical approach** |  |  |  |  |  |
| 1.1 Is a qualitative approach appropriate? | *appropriate* |  | *x* | *x* | *x* |
|  | *inappropriate* |  |  |  |  |
|  | *unsure* | *x* |  |  |  |
| 1.2 Is the study clear in what it seeks to do? | *clear* | *x* | *x* | *x* | *x* |
|  | *unclear* |  |  |  |  |
|  | *mixed* |  |  |  |  |
| **Study design** |  |  |  |  |  |
| 2.1 How defensible/rigorous is the study design and methodology? | *defensible* |  | x | x | x |
|  | *not defensible* |  |  |  |  |
|  | *unsure* | x |  |  |  |
| **Data collection** |  |  |  |  |  |
| 3.1 How well was data collection carried out? | *appropriate* |  | x | x | x |
|  | *inappropriate* |  |  |  |  |
|  | *unsure / unclear* | x |  |  |  |
| **Validity** |  |  |  |  |  |
| 4.1 Is researcher’s role clearly described? | *clear* | x |  |  | x |
|  | *unclear* |  |  | x |  |
|  | *not described* |  | x |  |  |
| 4.2 Is context clearly described? | *clear* |  | x | x | x |
|  | *unclear* | x |  |  |  |
|  | *unsure* |  |  |  |  |
| 4.3 Were methods reliable? | *reliable* |  | x | x | x |
|  | *unreliable* |  |  |  |  |
|  | *unsure* | x |  |  |  |
| **Analysis** |  |  |  |  |  |
| 5.1 Is data analysis sufficiently rigorous? | *rigorous* |  | x |  | x |
|  | *not rigorous* | X |  | x |  |
|  | *unsure / unreported* |  |  |  |  |
| 5.2 Are the data ‘rich’? | *rich* |  |  |  | x |
|  | *poor* | x | x | x |  |
|  | *poorly reported* | x |  |  |  |
| 5.3 Is the analysis reliable? | *reliable* |  | x |  | x |
|  | *unreliable* |  |  |  |  |
|  | *unsure / unreported* | x |  | x |  |
| 5.4 Are findings convincing? | *convincing* |  | x |  |  |
|  | *not convincing* |  |  |  | x |
|  | *unsure* | x |  | x |  |
| 5.5 Are findings relevant to aims of the study? | *relevant* | x | x | x | x |
|  | *irrelevant* |  |  |  |  |
|  | *part relevant* |  |  |  |  |
| 5.6 Are conclusions adequate? | *adequate* | x | x | x | x |
|  | *inadequate* |  |  |  |  |
|  | *unsure* |  |  |  |  |
| **Ethics** |  |  |  |  |  |
| 6.1 Clear & coherent reporting of ethical considerations? | *clear* | x |  | x | x |
|  | *unclear* |  |  |  |  |
|  | *unsure /unreported* | x |  |  |  |
| **Overall quality**: G=Good; M=Moderate P=Poor | | P | G | M | G |
