# Supplementary material for: Knowledge and Awareness of HPV Vaccine and Acceptability to Vaccinate in Sub-Saharan Africa: A Systematic Review
Source: PLoS One. 2014 Mar 11;9(3):e90912. doi: 10.1371/journal.pone.0090912 (PMC3949716; doi:10.1371/journal.pone.0090912)
Supplement: Table S2 — Quality assessment of surveys. (DOCX) [file pone.0090912.s003.docx]

**Supplementary Table 2. Quality assessment of surveys**

|  | Ayissi et a., 2012 | Becker-Dreps et al., 2010 | Binagwaho et al., 2012 | Coleman et al., 2011 | | DiAngi et al., 2011 | Francis et al., 2010 | Galagan et al., 2013 | Iliyasu et al., 2010 | Ladner et al., 2012 | LaMontagne et al., 2011 | Liu et al., 2012 | Makwe et al., 2011 | Mbamara et al., 2011 | McCarey et al., 2011 | Morhason-Bello 2009 | Moodley et al., 2013 | Mupepi et al., 2011 | Poole et al. 2012 | Rositch et al., 2012 | Ugwu et al., 2012 | Urasa and Darj 2011 | Wamai et al., 2012 | Wamai et al., 2013 | Watson-Jones et al., 2012a | Watson-Jones et al., 2012b |
| --- | --- | --- | --- | --- | --- | --- | --- | --- | --- | --- | --- | --- | --- | --- | --- | --- | --- | --- | --- | --- | --- | --- | --- | --- | --- | --- |
| **Sample Size** | ✓ | S | ✓ | | ✓ | ✓ | S | ✓ | ✓ | ✓ | ✓ | ✓ | S | S | ✓ | L | ✓ | ✓ | ✓ | ✓ | S | S | ✓ | S | ✓ | ✓ |
| **Response Rate** | ✓ | ✓ | ✓ | | ✓ | ✓ | ✓ | ✓ | ✓ | ✓ | ✓ | ✓ | ✓ | ✓ | ✓ | R | ✓ | ✓ | ✓ | ✓ | ✓ | ✓ | ✓ | ✓ | ✓ | ✓ |
| **Overall clarity of aims and method:** *is there sufficient detail and clarity; can the reader make sense of it?* | ✓ | ✓ | ✓ | | ✓ | ✓ | ✓ | ✓ | ✓ | ✓ | ✓ | ✓ | ✓ | ✓ | ✓ | ✓ | ✓ | ✓ | ✓ | ✓ | ✓ | ✓ | ✓ | ✓ | ✓ | ✓ |
| **Selection of the sample:** *could responders differ from non-responders, or from the background population?* | ✓ | ✓ | ✓ | | ✓ | ✓ | ✓ | ✓ | ✓ | ✓ | ✓ | ✓ | ✓ | ✓ | ✓ | ✓ | ✓ | ✓ | ? | ✓ | ✓ | ✓ | ✓ | ✓ | ✓ | ✓ |
| **Measurement issues:** *are the measures reported objective and reliable?* | ✓ | ✓ | ✓ | | ✓ | ✓ | ✓ | ✓ | ✓ | ✓ | ✓ | ✓ | ✓ | ✓ | ✓ | ✓ | ✓ | ✓ | ? | ✓ | ✓ | ✓ | ✓ | ✓ | ✓ | ✓ |
| **Survey methods:** *was the survey carried out in a trustworthy way?* | ✓ | ✓ | ✓ | | ✓ | ✓ | ✓ | ✓ | ✓ | ✓ | ✓ | ✓ | ✓ | ✓ | ✓ | ✓ | ✓ | ✓ | ✓ | ✓ | ✓ | ✓ | ✓ | ✓ | ✓ | ✓ |
| **Data & statistical issues:** *was the analysis appropriately conducted?* | ✓ | ✓ | ✓ | | ✓ | ✓ | ✓ | ✓ | ✓ | ✓ | ✓ | ✓ | ✓ | ✓ | ✓ | ✓ | ✓ | ✓ | ? | ✓ | ✓ | ✓ | ✓ | ✓ | ✓ | ✓ |
| **Bias:** *is there evidence of any other bias (e.g. funding bias)?* | ✓ | ✓ | ✓ | | ✓ | ✓ | ✓ | ✓ | ✓ | ✓ | ✓ | ✓ | ✓ | ✓ | ✓ | ? | ✓ | ✓ | ? | ✓ | ✓ | ✓ | ✓ | ✓ | ✓ | ✓ |

| ✓ = satisfactory  NR = not reported  ? = unclear | R = response rate not reported  L = low response rate < 40%  S = small sample< 200 | GSK = Glaxo Smith Klein (Cervarix™)  MF = Merck Foundation (Gardasil®) |
| --- | --- | --- |
