# Supplementary material for: Knowledge and Awareness of HPV Vaccine and Acceptability to Vaccinate in Sub-Saharan Africa: A Systematic Review
Source: PLoS One. 2014 Mar 11;9(3):e90912. doi: 10.1371/journal.pone.0090912 (PMC3949716; doi:10.1371/journal.pone.0090912)
Supplement: Appendix S1 — Search strategies for bibliographic databases. (DOCX) [file pone.0090912.s005.docx]

Appendix 1: Search strategies for bibliographic databases.

PubMed/MEDLINE (NLM)

("Attitude to Health"[mesh:noexp] OR "Health Knowledge, Attitudes, Practice"[mesh] OR "Patient Acceptance of Health Care"[mesh] OR accept[tiab] OR acceptance[tiab] OR acceptability[tiab] OR knowledge[tiab] OR awareness[tiab] OR belief*[tiab] OR attitude*[tiab] OR perception*[tiab] OR understanding*[tiab] OR adherence[tiab] OR compliance[tiab] OR uptake[tiab]) OR willingness[tiab]

AND

("Papillomavirus Vaccines"[mesh] OR (("Papillomavirus Infections"[mesh] OR "Papillomaviridae"[mesh]) AND ("Vaccination"[mesh] OR "Immunization"[mesh] OR "Immunization Programs"[mesh])) OR ((alphapapilloma*[tiab] OR betapapilloma*[tiab] OR gammapapilloma*[tiab] OR mupapilloma*[tiab] OR papilloma*[tiab] OR HPV*[tiab] OR wart virus[tiab] OR genital wart*[tiab]) AND (vaccine*[tiab] OR vaccination*[tiab] OR immuniz*[tiab] OR immunis*[tiab])) OR gardasil[tiab] OR cervarix[tiab])

AND

("Africa South of the Sahara"[Mesh] OR africa[all fields] OR southern africa[all fields] OR west africa[all fields] OR western africa[all fields] OR central africa[all fields] OR west african[all fields] OR east african[all fields] OR south african[tiab] OR central african[tiab] OR Cameroon[all fields] OR Central African Republic[all fields] OR Chad[all fields] OR Congo[all fields] OR Democratic Republic of the Congo[all fields] OR Equatorial Guinea[all fields] OR Gabon[all fields] OR Burundi[all fields] OR Djibouti[all fields] OR Eritrea[all fields] OR Ethiopia[all fields] OR Kenya[all fields] OR Rwanda[all fields] OR Somalia[all fields] OR Sudan[all fields] OR Tanzania[all fields] OR Uganda[all fields] OR Angola[all fields] OR Botswana[all fields] OR Lesotho[all fields] OR Malawi[all fields] OR Mozambique[all fields] OR Namibia[all fields] OR South Africa[all fields] OR Swaziland[all fields] OR Zambia[all fields] OR Zimbabwe[all fields] OR Benin[all fields] OR Burkina Faso[all fields] OR Cape Verde[all fields] OR Cote d'Ivoire[all fields] OR "ivory coast"[all fields] OR Gambia[all fields] OR Ghana[all fields] OR Guinea[all fields] OR Guinea-Bissau[all fields] OR Liberia[all fields] OR Mali[all fields] OR Mauritania[all fields] OR Niger[all fields] OR Nigeria[all fields] OR Senegal[all fields] OR Sierra Leone[all fields] OR Togo[all fields])

Embase (Elsevier)

('attitude to health'/exp OR 'patient attitude'/exp OR 'awareness'/exp OR 'knowledge'/de OR accept:ti,ab OR acceptance:ti,ab OR acceptability:ti,ab OR knowledge:ti,ab OR awareness:ti,ab OR belief*:ti,ab OR attitude*:ti,ab OR perception*:ti,ab OR understanding*ti,ab OR adherence:ti,ab OR compliance:ti,ab OR uptake:ti,ab OR willingness:ti,ab)

AND

('Wart virus vaccine'/exp OR (('papillomavirus infection'/exp OR 'Papilloma virus'/exp) AND 'immunization'/exp) OR ((alphapapilloma*:ti,ab OR betapapilloma*:ti,ab OR gammapapilloma*:ti,ab OR mupapilloma*:ti,ab OR papilloma*:ti,ab OR HPV*:ti,ab OR wart virus:ti,ab OR genital wart*:ti,ab) AND (vaccine*:ti,ab OR vaccination*:ti,ab OR immuniz*:ti,ab OR immunis*:ti,ab)) OR gardasil:ti,ab OR cervarix:ti,ab)

AND

('Africa south of the Sahara'/exp OR 'africa' OR 'southern africa' OR 'west africa' OR 'western africa' OR 'central africa' OR 'east african' OR 'west african' OR 'south african' OR 'central african' OR 'Cameroon' OR 'Central African Republic' OR 'Chad' OR 'Congo' OR 'Democratic Republic of the Congo' OR 'Equatorial Guinea' OR 'Gabon' OR 'Burundi' OR 'Djibouti' OR 'Eritrea' OR 'Ethiopia' OR 'Kenya' OR 'Rwanda' OR 'Somalia' OR 'Sudan' OR 'Tanzania' OR 'Uganda' OR 'Angola' OR 'Botswana' OR 'Lesotho' OR 'Malawi' OR 'Mozambique' OR 'Namibia' OR 'South Africa' OR 'Swaziland' OR 'Zambia' OR 'Zimbabwe' OR 'Benin' OR 'Burkina Faso' OR 'Cape Verde' OR 'Cote d Ivoire' OR 'Cote dIvoire' OR 'ivory coast' OR 'Gambia' OR 'Ghana' OR 'Guinea' OR 'Guinea-Bissau' OR 'Liberia' OR 'Mali' OR 'Mauritania' OR 'Niger' OR 'Nigeria' OR 'Senegal' OR 'Sierra Leone' OR 'Togo')

CINAHL (EBSCO)

(MH ("Attitude to Health+" OR "Patient Compliance+" OR "Health Knowledge" OR "Patient Attitudes") OR TI (accept OR acceptance OR acceptability OR knowledge OR awareness OR belief* OR attitude* OR perception* OR understanding* OR adherence OR compliance OR uptake OR willingness) OR AB (accept OR acceptance OR acceptability OR knowledge OR awareness OR belief* OR attitude* OR perception* OR understanding* OR adherence OR compliance OR uptake OR willingness))

AND

(MH ("Papillomavirus Vaccine" OR (("Papillomavirus Infections" OR "Papillomaviruses") AND ("Immunization+" OR "Immunization Programs"))) OR TI (((alphapapilloma* OR betapapilloma* OR gammapapilloma* OR mupapilloma* OR papilloma* OR HPV* OR wart virus OR genital wart*) AND (vaccine* OR vaccination* OR immuniz* OR immunis*)) OR gardasil OR cervarix) OR AB (((alphapapilloma* OR betapapilloma* OR gammapapilloma* OR mupapilloma* OR papilloma* OR HPV* OR wart virus OR genital wart*) AND (vaccine* OR vaccination* OR immuniz* OR immunis*)) OR gardasil OR cervarix))

AND

(TX (africa OR "southern africa" OR "west africa" OR "western africa" OR "west african" OR "east african" OR "south african" OR "central africa" OR "central african" OR Cameroon OR "Central African Republic" OR Chad OR Congo OR "Equatorial Guinea" OR Gabon OR Burundi OR Djibouti OR Eritrea OR Ethiopia OR Kenya OR Rwanda OR Somalia OR Sudan OR Tanzania OR Uganda OR Angola OR Botswana OR Lesotho OR Malawi OR Mozambique OR Namibia OR "South Africa" OR Swaziland OR Zambia OR Zimbabwe OR Benin OR "Burkina Faso" OR "Cape Verde" OR "Cote d'Ivoire" OR Gambia OR Ghana OR Guinea OR "Guinea-Bissau" OR Liberia OR Mali OR Mauritania OR Niger OR Nigeria OR Senegal OR "Sierra Leone" OR Togo))

POPLINE (K4Health)

(accept OR acceptance OR acceptability OR knowledge OR awareness OR belief* OR attitude* OR perception* OR understanding* OR adherence OR compliance OR uptake OR willingness)

AND

(((alphapapilloma* OR betapapilloma* OR gammapapilloma* OR mupapilloma* OR papilloma* OR HPV* OR "wart virus" OR "genital wart*") AND (vaccine* OR vaccination* OR immuniz* OR immunis*)) OR gardasil OR cervarix)

AND

((africa OR "southern africa" OR "west africa" OR "western africa" OR "central africa" OR "west african" OR "east african" OR "south african" OR "central african" OR Cameroon OR "Central African Republic" OR Chad OR Congo OR "Democratic Republic of the Congo" OR "Equatorial Guinea" OR Gabon OR Burundi OR Djibouti OR Eritrea OR Ethiopia OR Kenya OR Rwanda OR Somalia OR Sudan OR Tanzania OR Uganda OR Angola OR Botswana OR Lesotho OR Malawi OR Mozambique OR Namibia OR South Africa OR Swaziland OR Zambia OR Zimbabwe OR Benin OR "Burkina Faso" OR "Cape Verde" OR "Cote d'Ivoire" OR "ivory coast" OR Gambia OR Ghana OR Guinea OR "Guinea-Bissau" OR Liberia OR Mali OR Mauritania OR Niger OR Nigeria OR Senegal OR Sierra Leone OR Togo))

AIM (WHO)

(accept OR acceptance OR acceptability OR knowledge OR awareness OR belief OR beliefs OR attitude OR attitudes OR perception* OR understanding OR adherence OR compliance OR uptake OR willingness)

AND

(((papilloma OR papillomavirus OR HPV) AND (vaccine OR vaccines OR vaccination OR vaccinations OR immunization OR immunizations OR immunisation OR immunisations)) OR gardasil OR cervarix
